# Outcomes and resource use for over 80 year olds admitted to a UK critical care unit after an emergency laparotomy over a 3-year period

**DOI:** 10.1186/cc11010

**Published:** 2012-03-20

**Authors:** V Banks, C Scott

**Affiliations:** 1 Northern General Hospital, Sheffield, UK

## Introduction

There are few data on older people emergency surgical critical care (CC) admissions and the potential implications for future resource demands and service planning.

## Methods

Retrospective data were collected from a cohort of patients >80 years old admitted after emergency surgery between 2009 and 2011. CC and hospital information databases were used. Data included mortality, length of stay (LOS) and duration of renal replacement therapy (RRT) and advanced respiratory support (ARS).

## Results

A total of 118 patients were admitted; 52% female: mean age 85 years, male mean age: 84 years. In total, 69% were general surgical, 22% vascular, and 9% hepatobiliary. Eleven per cent required RRT for a mean of 3.6 days, and 41% needed ARS for a mean of 3.8 days. See Table 1.

**Table 1:** 

Patient group	CC mortality, *n *(%)	Hospital mortality, *n *(%)	LOS CC (days)	LOS hospital (days)
All patients, *n *= 118	20 (17%)	39 (33%)	1 to 34, 4.5 mean	1 to 247, 29 mean
Age 80 to 84, *n *= 70	15 (21%)	29 (41%)	1 to 34, 4.9 mean	1 to 247, 28 mean
Age >85, *n *= 48	5 (10%)	10 (21%)	1 to 32, 4.1 mean	1 to 171, 31 mean
ARS, *n *= 49	15 (30%)	22 (45%)	1 to 34, 7.5 mean	1 to 79, 20 mean
No ARS, *n *= 69	5 (7%)	17 (25%)	1 to 24, 3.8 mean	1 to 247, 35 mean
RRT, *n *= 13	7 (54%)	9 (69%)	1 to 34, 8 mean	1 to 247, 35 mean
No RRT, *n *= 105	13 (12%)	30 (29%)	1 to 32, 4 mean	1 to 247, 30 mean

## Conclusion

CC and hospital mortality was 17% and 33% respectively. This study concurs with another which demonstrated that age is not a good predictor of outcome post surgery [1]. These patients did not have a significant impact on RRT or ARS resources or CC LOS.
